# COVID’s long shadow: How SARS-CoV-2 infection, COVID-19 severity, and vaccination status affect long-term cognitive performance and health

**DOI:** 10.1093/biomethods/bpad038

**Published:** 2023-12-07

**Authors:** Jaroslav Flegr, Ashkan Latifi

**Affiliations:** Department of Philosophy and History of Sciences, Faculty of Science, Laboratory of Evolutionary Biology, Charles University, Prague 128 00, Czechia; Department of Philosophy and History of Sciences, Faculty of Science, Laboratory of Evolutionary Biology, Charles University, Prague 128 00, Czechia

**Keywords:** SARS-CoV-2, COVID-19, cognitive performance, cognition, mental health, long-term effects

## Abstract

COVID-19 affects a variety of organs and systems of the body including the central nervous system. Recent research has shown that COVID-19 survivors often experience neurological and psychiatric complications that can last for months after infection. We conducted a large Internet study using online tests to analyze the effects of SARS-CoV-2 infection, COVID-19 severity, and vaccination on health, intelligence, memory, and information processing precision and speed in a cohort of 4445 subjects. We found that both SARS-CoV-2 infection and COVID-19 severity were associated with negative impacts on patients’ health. Furthermore, we observed a negative association between COVID-19 severity and cognitive performance. Younger participants had a higher likelihood of SARS-CoV-2 contraction, while the elderly had a higher likelihood of severe COVID-19 and vaccination. The association between age and COVID-19 severity was primarily mediated by older participants’ impaired long-term health. Vaccination was positively associated with intelligence and the precision of information processing. However, the positive association between vaccination and intelligence was likely mediated by achieved education, which was itself strongly associated with the likelihood of being vaccinated.

## Introduction

SARS-CoV-2 appeared in Wuhan, China in December 2019. Although this viral infection was initially deemed to be a respiratory disease, researchers have since studied and discovered other aspects of its pathogenicity. For example, it is argued that COVID-19 is also a neuroinvasive disease that, by triggering a cytokine storm, can affect the nervous system and may be potentially involved in the onset and progression of neuropsychiatric and neurodegenerative complications [1]. These adverse effects of COVID-19 are not limited to the immediate symptoms of the infection but also include post-infection sequelae affecting mental and physical health, cognition, and fatigue. Notably, some of these aspects, specifically mental health and fatigue, may even begin to deteriorate again after a long period of improvement [2]. A comprehensive review of the neuropsychiatric pathogenicity of long COVID reported that common complications included fatigue, cognitive impairment, sleep disorders, depression, anxiety, and post-traumatic stress disorder [3].

A substantial body of research, including [4], has established a connection between impairment of cognitive functions and brain inflammation. Accordingly, among other concerns, researchers have begun to investigate the possible COVID-induced cognitive impairment in individuals who contracted the disease. A study investigating cognitive functioning in 54 patients who had mild COVID-19 and 36 matched healthy controls, indicated worse attention, precision, and slower information processing in the patient group than the control group; in addition, a decline in attention and short-term memory was observed in the patient group compared with the controls [5]. Regarding broader aspects of cognition, a study with a sample of 32 males and 182 females with a history of COVID-19 implementing tests for processing speed, attention, verbal, visual, prospective and working memory, executive functions, visuospatial skills, and language showed that more than 85% of the subjects had alterations in at least one of the tests [6]. Another study using a comprehensive collection of tests reported that global cognitive functions, memory functions, visual perceptual functions, and neuropsychiatric status, but not attention functions and executive functions (except for Phonemic fluency and TMT-A), were negatively affected by COVID-19 in the patients when compared with controls [7].

As the aforementioned studies suggest, COVID-19 can negatively affect cognitive functions and health in patients with a history of COVID. However, most of these studies were based on small sample sizes and had somewhat controversial findings. For example, although Zhou *et al*. [8] found no significant difference between the patient group and controls in cognitive processing, executive functions, memory, concentration, and resistance to information interference, and they observed lower reaction times in the patient group, Demir *et al*. [5] reported higher reaction times, slower processing speed, and lower resistance to Stroop interference in their patient group compared with controls. Similarly, among other findings, processing speed, attention, working memory, and executive functions were found to be more compromised in patients in another study [6]; however, other research did not find a significant difference between the patients and controls in attention and executive functions [7]. The role of age has also proved controversial, as one study reported a negative association between age and impairment in cognitive functioning [6], while another study found a positive association in the patient group [9]. Findings regarding COVID severity have also been somewhat controversial, as one study found no significant relationship between disease severity and cognitive deficits [7], while other studies reported significant associations [10]. Furthermore, the possible interrelationships of age, long-term sickness, current sickness, and COVID vaccination with cognition have not been addressed in previous research.

With the objective of addressing these gaps in knowledge, the current cross-sectional study sought to investigate the impact of SARS-CoV-2 infection, COVID-19 severity, and COVID-19 vaccination on the cognitive performance and health outcomes of a cohort of 4445 participants. We measured intelligence, memory, reaction times, and information processing speed using a set of four online tests, and then analyzed the data using partial Kendall Tau correlation tests and path analysis techniques.

## Materials and methods

### Participants

An electronic survey consisting of several performance tests and questionnaires, with only some related to the present study, was advertised on Facebook and Twitter as a project “studying the interconnections between moral attitudes, cognitive performance, and various biological, psychological, and sociodemographic factors.” On the first page of the first questionnaire, participants were informed that the study was anonymous and that they could discontinue their participation at any time. They were also given the following information: “We will be determining which biological and psychological characteristics influence the results of performance tests and moral attitudes. We will measure your memory, speed, ability to concentrate, and intelligence.” Only those who confirmed they were over 15 years old and provided informed consent by clicking the corresponding button were allowed to participate in the study. The study was partially or fully completed by 8,800 subjects between March and June 2022. The project, including the method of obtaining informed consent, was approved by the Institutional Review Board of the Faculty of Science, Charles University (No. 2021/4), and all methods were performed in accordance with the relevant guidelines and regulations.

### Questionnaires and tests

The survey consisted of several electronic performance tests and a series of questionnaires, of which only some were related to this project. The total time for completing the questionnaire was between 40 and 60 min for most participants.

In the survey, we assessed the intelligence of the subjects using the Cattel 16PF test (Variant A, Scale B) [11] and their memory with a modified Meili test [12, 13]. During the Meili test, participants were first shown 12 words (knife, frog, pump, chain, tree, collar, ice, glasses, arrow, train, bars, and rifle) for 24 s and then, about 30 min into the survey, they were asked to recall these words from a list of 24. Psychomotor performance (reaction time and precision) was measured using the Stroop test. This version of the Stroop test consisted of three parts with breaks for instructions and rest in between. In Part A, participants had to select a specific word (e.g. “red”) from a set of four words (“red,” “green,” “blue,” and “brown”) displayed in the same order in the center of the screen. The words were written in a font color that did not match their meaning. The command specifying which word to select was written in the upper part of the screen and participants were instructed to ignore the font color. In Part B, the stimuli were the same, but participants were asked to select a word written in a specific color, ignoring the meaning of the displayed words. Part C was similar to Part A, but the command specifying which word to select was always written in a different color that did not match either the meaning or color of the displayed stimuli. Before each part, participants received instructions on the rules, were informed of how many times the test would run (always five times), and were asked to react as quickly as possible. Participants could start each part of the Stroop test by pressing the “Start test” button. A similar test was utilized to measure reaction times. Participants were instructed to press a button corresponding to a specific character, which included the letters A, B, C, and D. The characters were presented in the same order and all appeared in the same color (black). This variant of the test was administered eight times at the beginning of the experiment.

In the anamnestic part of the questionnaire, participants answered 11 questions about their long-term physical health. They had to respond to the questions about frequencies of doctor visits, fatigue, headaches, other physical pain, neurological diseases, and other chronic physical issues using 6-point ordinal scales. They also had to report the number of nonmental health medications prescribed by a doctor they were currently using (10 meant 9 or more), and the number of times they spent more than a week in the hospital in the past 5 years (5 meant 6 or more times). Participants were also asked about their usual physical feeling (5-point scale). This question was asked two times, once at the beginning of the questionnaire and once near its end, that is about 30 min later. Finally, they were asked how many years they expected to live (1: more than 99, 2: 90–99, 3: 80–89, 4: 70–79, 5: 60–69, 6: less than 60).

In addition to rating their typical physical state, participants also rated their current physical state on a 5-point scale at the beginning and toward the end of the questionnaire. Indices for long-term physical problems (long-term sickness) and current physical health issues (current sickness) were calculated as the mean *Z*-scores of the relevant questions [14].

In the anamnestic part of the survey, participants were also asked about their age, sex (men coded as 1, women coded as 0), education [ordinal scale 1–10: 1, Basic education only; 2, Basic education plus studying at secondary school; 3, Secondary education including vocational training (without A-levels); 4, Complete secondary education or higher vocational training (A-levels or diploma); 5, Complete secondary education or higher vocational training, plus studying for a bachelor’s degree; 6, Bachelor’s degree (BA, BSc); 7, Studying for master’s degree; 8, Master’s degree (MA, MSc, MBA, MD, LL.M, MEng, etc.); 9, Master’s degree, plus studying for a doctoral degree; 10, Doctoral degree (PhD, DPhil, EdD, etc.)] and if they had been infected with SARS-CoV-2 (had COVID) (1: “not yet,” 2: “yes, I was diagnosed with COVID,” 3: “probably yes, but I was not diagnosed with COVID,” 4: “I am waiting for the result of a diagnostic test,” 5: “No but I was in quarantine”). For purposes of this study, answers 1 and 5 were coded as 0 (COVID-negative), answer 2 as 1 (COVID-positive), and answers 3 and 4 were coded as NA (data not available) as these participants’ COVID-19 infection history could not be reliably ascertained. If they answered “yes, I was diagnosed with COVID,” they were asked for the start and end dates of their COVID illness and to rate its severity on a 6-point scale (1: “No symptoms,” 2: “Like mild flu,” 3: “Like normal flu,” 4: “Like severe flu,” 5: “I was hospitalized,” and 6: “I was treated at an Intensive Care Unit”). The participants were also asked if they had been vaccinated against COVID (binary variables 0/1; we did not discriminate how many doses they received).

### Data analyses

The influence of infection, COVID severity and duration, time since COVID onset, and vaccination status on health and cognitive performance were assessed using nonparametric partial Kendall correlation tests. These tests were run using the R script Explorer version 1.0 [15], which utilizes the ppcor R package [16]. In the analyses of the mixed sample of men and women, we controlled for both age and sex, while in the separate analyses for men and women, we controlled only for age. The Kendall correlation test allows for controlling confounding variables and is robust against the presence of outliers and the distribution shape of variables in general. To account for multiple testing, we controlled the effects using the Benjamini–Hochberg procedure, with a false discovery rate set at 0.10 [17]. Path analysis (PA) was conducted using lavaan version 0.6.12 [18] and semPlot 1.1.6 [19]. The dataset is publicly available on Figshare [20].

### Technical notes

Throughout the article, the term “effect” is used in a statistical sense, meaning an observed association—the difference between the true population parameter and its null hypothesis value. Only in the “Discussion” section do we distinguish between cause and effect. Some parts of this study had a confirmatory, others an exploratory character. We, therefore, report the results corrected and noncorrected for multiple tests and discuss not only the formally significant effects but also trends that were not formally significant.

## Results

### Dataset description

**Figure 1.**
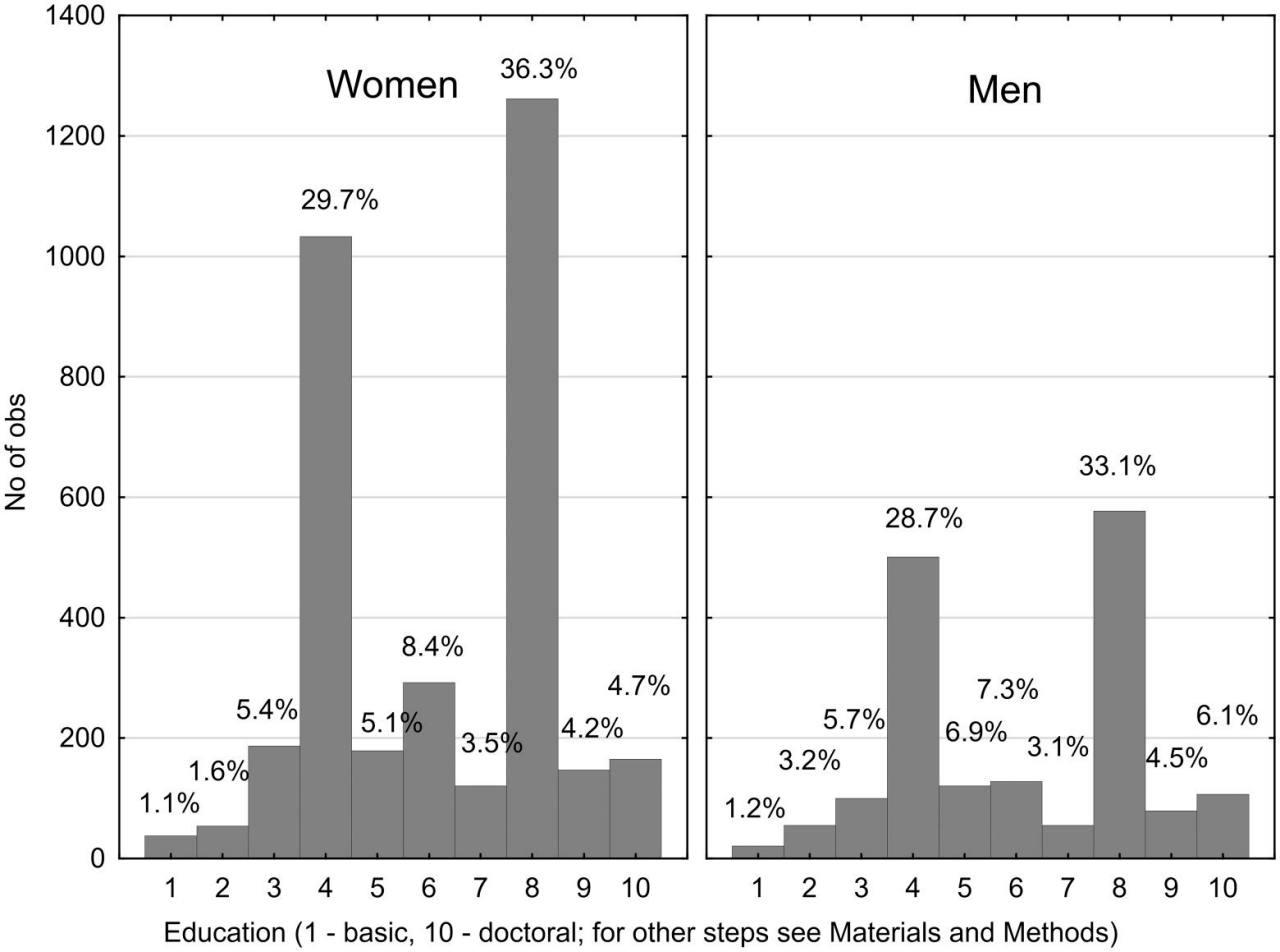
Education of female and male participants of the study. The codes 4 and 8 correspond to Complete secondary education or higher vocational training (A-levels or diploma), and master’s degree, respectively.

**Figure 2.**
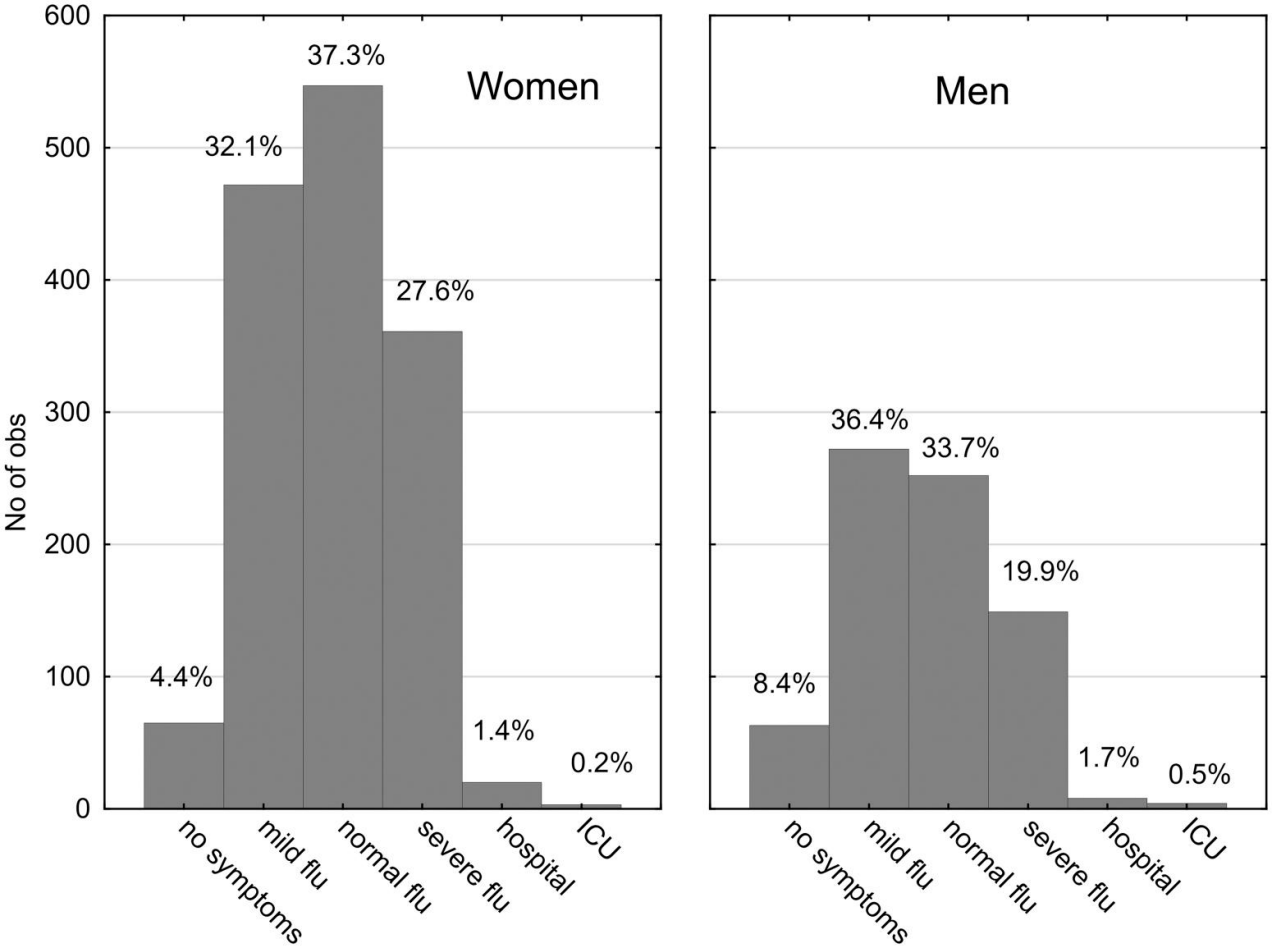
Severity of the course of COVID-19 in the female and male participants of the study. Codes 1 to 6 correspond to “No symptoms,” “Like mild flu,” “Like normal flu,” “Like severe flu,” “hospitalized,” and “at ICU,” respectively.

**Figure 3.**
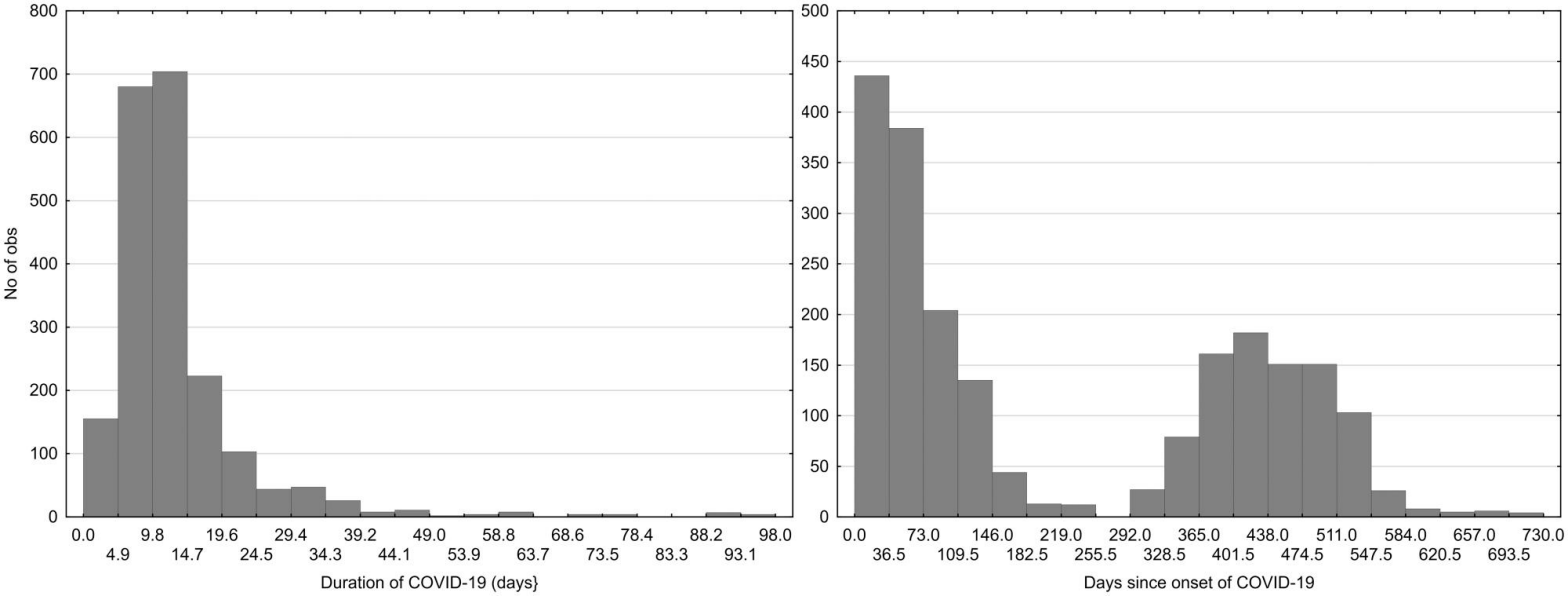
Duration of COVID-19 and time passed since start of the COVID. The left histogram does not show 21 (1.02%) of the participants, including 17 (1.26%) women and 4 (0.56%) men, who reported a duration of COVID longer than 100 days.

The initial dataset comprised 5230 participants who answered a question about their COVID status (66% women and 33% men). Out of these, 1793 had not yet been infected, 2246 had been diagnosed with COVID-19, 763 were possibly infected but not diagnosed, 22 were awaiting test results, and 406 had not been diagnosed but were in quarantine. Those who answered “No” or “No, but I was in quarantine” were categorized as COVID negative (coded 0), while those who answered “Yes, I was diagnosed with COVID” were categorized as COVID positive (coded 1). Data from other subjects were excluded from the dataset (785 participants). Therefore, the final dataset included 2199 COVID-negative and 2246 COVID-positive participants (4445 participants in total). Table 1 and Figs 1–3 present the descriptive statistics for the dataset.

**Table 1.** Descriptive statistics of the final set.

	Sex		COVID status		Vaccine	
	Women	Men	No	Yes	No	Yes
N	3485	1746	2195	2242	427	4777
Mean age (years)	42.94	39.74	43.40	40.60	39.05	42.11
Education	6.15	6.07	6.16	6.13	5.17	6.21
Long-term sickness	0.05	−0.09	0.02	−0.02	−0.05	0.00
Current sickness	0.07	−0.12	−0.03	0.02	0.07	0.00
Intelligence	9.05	9.54	9.15	9.28	8.77	9.26
Memory	7.75	7.39	7.54	7.71	7.67	7.63
Time Stroop test (sec.)	1.75	1.76	1.78	1.72	1.76	1.75
Precision Stroop test (score)	13.40	13.66	13.50	13.50	13.18	13.52
Reaction time (sec.)	1.17	1.13	1.18	1.13	1.15	1.16

Indices assessing long-term and current sickness were computed as mean *Z*-scores based on relevant health variables. Intelligence was assessed by the number of correct responses on the Cattell IQ test (Variant A, Scale B, with a maximum score of 12), memory was measured by the number of correctly identified items on the modified Meili test used in this study (with a maximum score of 12), and education was evaluated by a score on a 10-point ordinal scale (ranging from 1 for elementary education to 10 for a PhD or ThD).

The study’s findings revealed that a higher percentage of men (52.32%) were diagnosed with COVID compared to women (49.66%), but this difference did not reach statistical significance (OR = 1.11, 95 CI 0.97–1.26, *P* = .0969). Additionally, a significantly higher proportion of men (93.95%) reported being vaccinated compared to women (90.71%) (OR = 1.58, 95% CI 1.26–2.01, *P* = 6.042E-05). The study also demonstrated that vaccinated individuals were less likely to have been diagnosed with COVID (49.05%) compared to those who were not vaccinated (67.24%), with a highly significant difference (OR = 0.469, 95% CI 0.36–0.59, *P* = 7.366E-11). Although vaccinated subjects had a lower risk of hospitalization (1.21% versus 1.72%) and treatment in an Intensive Care Unit (0.25% versus 0.86%), the effect of vaccination on the course of the disease was not significant (Spearman *R* = −0.01, *P* = .64), likely due to the small number of nonvaccinated subjects and the low number of subjects with a severe course of COVID (Table 2).

**Table 2.** Course of COVID reported by vaccinated and nonvaccinated participants.

	Vaccinated	No symptoms	Mild flu	Normal flu	Severe flu	Hospital	ICU
Count	No	11	73	96	47	4	2
Percent		4.72	31.33	41.20	20.17	1.72	0.86
Count	Yes	117	670	703	462	24	5
Percent		5.91	33.82	35.49	23.32	1.21	0.25
Count	All	128	743	799	509	28	7
Percent		5.78	33.56	36.09	22.99	1.26	0.32

Effects of COVID-19-associated variables on health and performance: multivariate nonparametric analyses.

The initial analysis using *F*-tests and inspection of histograms indicated that the variances also differed between infected and noninfected subjects and between men and women. Given this and also because of the strong effects of age and sex on performance and health, a multivariate nonparametric test, a partial Kendall correlation test controlled for age and sex, was used to examine the associations between SARS-CoV-2 infection, course of COVID, and vaccination with the performance and health of subjects (Table 3).

**Table 3.** Correlation between COVID-related variables with age, sex, health, and performance.

		Partial Kendall Tau			
	Infected	Course	Duration of COVID	Since COVID	Vaccinated
Age	−**0.080**	**0.051**	**0.097**	−0.007	**0.060**
Sex	0.017	−**0.074**	−**0.052**	**0.033**	**0.061**
Education	−0.005	−**0.039**	−**0.042**	0.003	**0.111**
Long−term sickness	−0.017	**0.148**	**0.118**	−0.002	**0.031**
Current sickness	**0.023**	**0.122**	**0.097**	−**0.032**	−0.012
Intelligence	0.018	−0.035^*^	−0.020	0.014	0.064^*^
Memory	**0.020**	−0.043^*^	−0.009	−0.000	0.008
Precision Stroop test	−0.000	−0.031^*^	−0.008	0.007	0.048^*^
Time Stroop test (sec.)	−0.009	0.022	**0.031**	−0.018	−0.022^*^
Reaction time (sec.)	−0.040^*^	0.025^*^	0.021	−0.027	−0.013

			**P**-**values**		

Age	1.02E-15	0.000254	3.10E-11	0.614952	6.56E-11
Sex	0.088933	1.28E-04	0.000363	0.021335	2.84E-11
Education	0.578293	0.005089	0.00382	0.798982	2.83E-33
Long-term sickness	0.085133	1.18E-25	1.08E-15	0.839863	0.000699
Current sickness	0.017378	7.01E-18	3.32E-11	0.025363	0.178982
Intelligence	0.065577	0.012089	0.169031	0.323175	3.64E-12
Memory	0.042148	0.002362	0.525511	0.986622	0.373842
Precision Stroop test	0.962318	0.028728	0.544026	0.586288	1.95E-07
Time Stroop test (sec.)	0.323233	0.113122	0.030953	0.192413	0.015192
Reaction time (sec.)	5.30E-05	0.074332	0.145262	0.057141	0.141735

Sex was a binary variable, with women coded as 0 and men coded as 1. Negative Tau values, for example, indicate that women reported a more severe COVID course than men. Infected and vaccinated were binary variables (No: 0, Yes: 1), and the course of COVID was rated on a 6-point scale (1: no symptoms, 6: treated at the Intensive Care Unit). The duration of COVID was measured in days from the start to the end of COVID, and the time since COVID was measured in days from the start of COVID to completing the internet tests. Significant effects are printed in bold. Asterisks indicate effects that remain significant after a correction for multiple tests; this correction was applied exclusively to cognitive performance tests, not to other variables in the table.

### Path analysis of direct and indirect effects of SARS-CoV-2 infection

**Figure 4.**
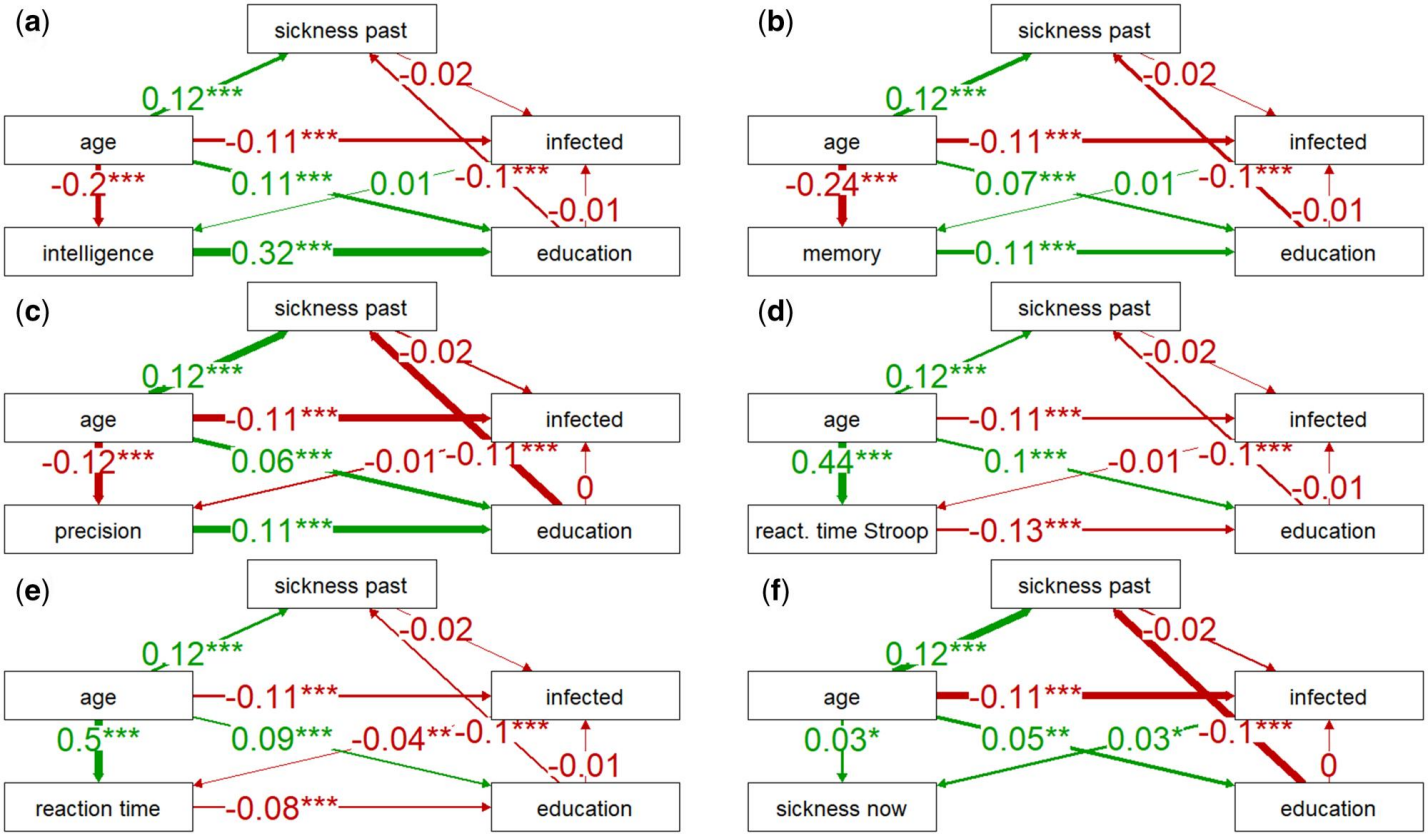
Correlation of being infected with SARS-CoV-2 with health and cognitive performance. Schemes a–e show the direct and indirect effects (path coefficients) of health and COVID-related variables on cognitive performance. The scheme f shows similar effects on the current sickness of the participants. The number of asterisks (one, two, or three) indicates their significance (0.05, 0.01, and 0.001, respectively). A positive path coefficient (green arrow) indicates that, for example, older participants reported a more severe COVID-19 course than younger participants.

**Figure 5.**
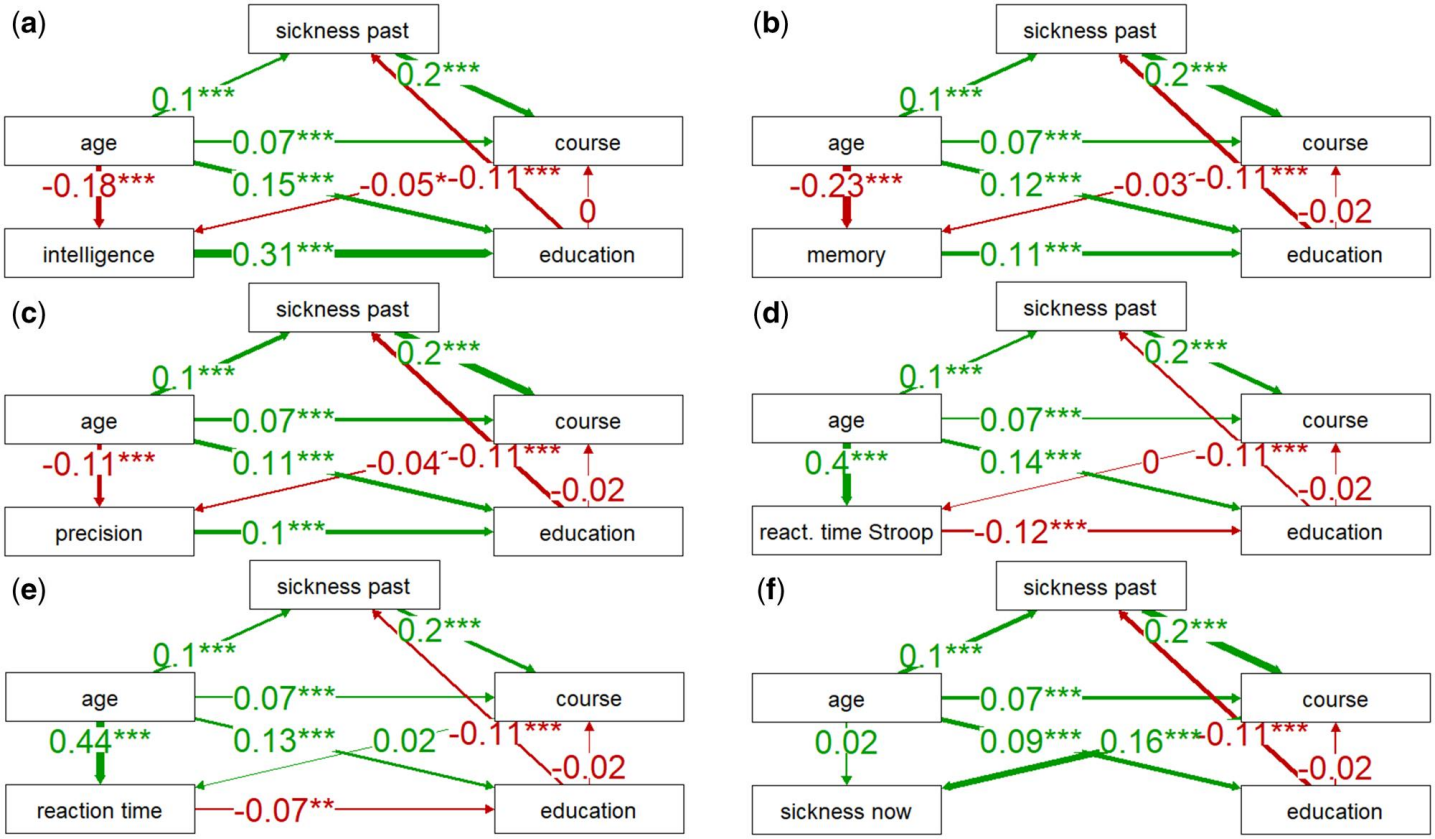
Correlation of COVID-19 severity with health and cognitive performance. For legend, see Fig. 4.

**Figure 6.**
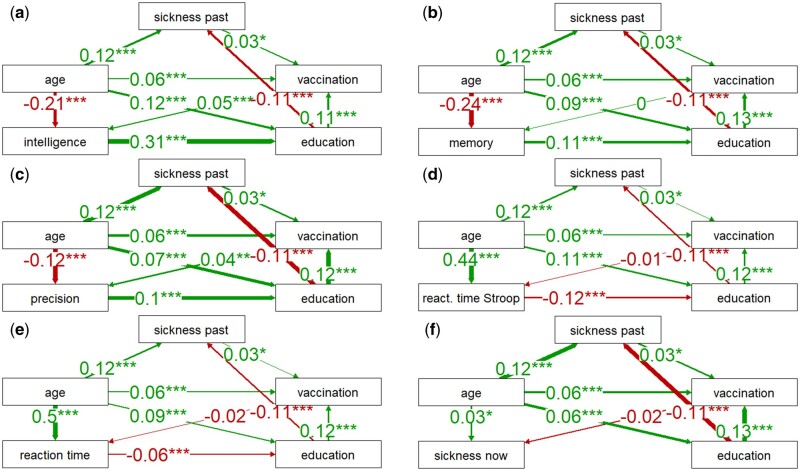
Correlation of vaccination and long-term sickness with current sickness and cognitive performance. For legend, see Fig. 4.

Correlation tests cannot distinguish between direct and indirect effects. The association between COVID and education is unlikely to be a result of COVID impacting education. However, the connection between COVID and IQ is not as clear-cut. To address these uncertainties, we used PA. The findings from the analyses are presented in Figs 3–6. To simplify the information and prevent redundancy, we have combined the verbal presentation of the results from PA with their interpretation in the following section.

The results of the PA for the binary variable infection (Fig. 4), specifically the comparison of path coefficients, indicated that the direct link between intelligence and infection was very weak and nonsignificant (0.01), but the positive effects of intelligence (0.32), memory (0.11), and precision (0.11), and negative effects of speed of information processing measured with the Stroop (−0.09) and simple reaction time tests (−0.09), and current sickness (−0.05) on achieved education were relatively strong and significant. Additionally, achieved education had a significant and negative effect on long-term sickness (−0.11), and long-term sickness may have had a slight negative effect on the risk of COVID infection (−0.02, not significant). This negative correlation may reflect the efforts of people with known health risks to avoid infection. The results also revealed that infection had a positive effect on current sickness (0.03). As to age, it had positive and significant effects on education (0.05–0.11), long-term sickness (0.12), reaction times (0.31–0.46), and current sickness (0.03) and significant and negative effects on IQ (−0.2), memory (−0.24), precision (−0.12), and infection (−0.11).

### Path analysis of direct and indirect effects of COVID-19 severity

PA produced clearer results for the ordinal variable representing the severity of COVID (Fig. 5). The analyses showed once again that the positive influences of intelligence (0.31), memory (0.11), and precision (0.1), and the negative influences of the speed of information processing measured with the Stroop (−0.11) and simple reaction time tests (−0.07), and current sickness (−0.04, not significant) on achieved education were also relatively strong. Nevertheless, the analyses also revealed that long-term sickness increased the likelihood of a severe COVID course (0.2), and that a severe course was associated with worse cognitive test performance except for reaction times (IQ: −0.05, memory: −0.03, precision of information processing: −0.04). However, only the effect on IQ was significant (IQ: *P* = .036, memory: *P* = .126, precision: *P* = .100). A severe course of COVID-19 had a strong adverse effect on current sickness (0.16, *P* < .01). These results indicated that the negative association between education and the course of COVID-19 detected by partial Kendall tests was mediated by the negative effect of education on long-term sickness and the positive effect of long-term sickness on COVID severity. The effect of COVID severity on cognitive test performance as well as current sickness, was most likely direct. In addition, age had positive and significant effects on long-term sickness (0.1), course (0.07), education (0.10–0.15), reaction times (0.36–0.38), and current sickness (0.02, not significant though). The effect of age on the course of disease was also indirectly mediated by long-term sickness suggesting that as the individual becomes older their history of health issues negatively affects the course of COVID-19 and makes it more likely for them to develop the severe form of the disease. Age again had a significant and negative effect on performance in cognitive tests (intelligence, −0.18; memory, −0.23; precision, −0.11; reaction times, 0.38 and 0.36).

### Path analysis of direct and indirect effects of COVID-19 vaccination

The results of the PA for the binary variable vaccination are presented in Fig. 6. The analysis showed that there was a significant positive correlation between poor health (Long-term sickness) and the likelihood of being vaccinated (0.03), supporting the idea that individuals who are aware of their health problems are more likely to try to avoid infection through vaccination. Additionally, higher levels of education were strongly correlated with a higher likelihood of being vaccinated (0.11–0.13). Being vaccinated also correlated positively with performance in cognitive tests, except for memory, but only the correlations with intelligence (0.05) and precision measured with the Stroop test (0.04) were significant. Moreover, being vaccinated was negatively correlated with current sickness (−0.02), but the effect was not significant (*P* = .190). It is possible that this correlation is due to the lower probability of a severe course of COVID-19 in vaccinated individuals. The correlations of long-term sickness, vaccination, education, reaction times, current sickness, and performance in cognitive tests with age also conceptually yielded the same results as those reported in Fig. 5.

## Discussion

Our cross-sectional study involved 2246 volunteers who had contracted COVID-19 and 2199 who had not. The study revealed that the infection itself had a weak but notable negative effect on the subject’s current health and a negligible effect on participants’ performance in cognitive tests. Similarly, the elapsed time since contracting the disease had an unexpectedly minimal influence on health and cognitive performance outcomes, with all changes being minor and statistically insignificant, except for a noted improvement in physical health. In contrast, the severity of COVID-19 had a negative effect on current health that was approximately five times stronger than the sole infection and a significant negative effect on intelligence but not performance in other cognitive tests. Vaccination showed no impact on current health status; yet, it was positively associated with better performance in cognitive tests measuring intelligence and precision. This suggests that individuals with higher cognitive abilities are more likely to opt for vaccination compared to those with lower cognitive performance.

Our findings also demonstrated that age had a positive and significant effect on being vaccinated and the severity of COVID and a negative and significant effect on catching the disease. The positive effects of age on vaccination and COVID severity were also positively and significantly mediated by long-term sickness.

### Outcomes of enduring COVID-19

The negative effect of COVID-19 on current health found in our study was in agreement with the findings of the studies that reported similar adverse effects on COVID-19 patients’ current health [21–25]. These negative effects even months after recovery may be the result of direct tissue damage afflicted by the virus on the host cells via its entry receptor ACE2, autoreactive T-cells causing immune system dysregulation, SARS-CoV-2-specific CD8 T-cell response, COVID-related cardiovascular and pulmonary complications, and/or neuroinflammation [26, 27]. Our results also showed that the effects of mere COVID-19 infection on cognitive performance and functions were weak; a finding that was in disagreement with the former studies that reported significant adverse effects of both mild and acute COVID-19 on cognition [5, 6, 28, 29]. This finding was somewhat in agreement with Zhou *et al*. [8] who reported no significant difference between their patient group and controls in cognitive functions except for continuous and selective attention, and also another study that found no significant difference between the patient group and controls in terms of cognition [30]. Many of the studies that reported impairment in cognitive performance and functions recruited subjects with a history of acute COVID-19 (e.g. [31, 32]. However, the binary classification of participants into “infected” and “non-infected” groups might have obscured the nuanced effects of disease severity on cognitive outcomes, possibly explaining the nonsignificant results. Nevertheless, we did account for variations in COVID severity in subsequent analyses.

Regarding age, it was negatively and significantly associated with infection and cognitive functions (intelligence, memory, and precision) and positively and significantly with reaction times. The negative correlation between age and risk of contracting COVID suggests that the older adults were presumably more cautious of this disease because of their subjective perceived vulnerability to it, the news on higher rates of mortality and COVID complications in the elderly, or both. This indicates that behavioral immunity, that is the ability to avoid infection, was possibly a relatively effective method of combating COVID-19, at least before the emergence of more contagious variants of the SARS-CoV-2 virus. These findings, regarding age and cognition, were in agreement with those studies that reported cognitive impairment in older adult COVID patients [33–35].

### Severity-specific consequences of COVID-19

Our analyses further suggested that COVID severity significantly negatively correlated with intelligence in the patient group. In addition, the performance measured as reaction times, precision, and memory were also negatively (but non-significantly) correlated with the severity of the disease; the negative effect of the severity of the disease on the performance in all five tests suggests that the total effect is statistically significant (*P* = .031, Fisher’s exact test). Therefore, our results align with studies indicating that severe COVID has a negative impact on cognitive performance [2, 10] and contradict those suggesting the opposite [7].

We also detected a strong negative effect of COVID severity on current health, which was in agreement with previous findings [22, 36]. This is also likely to be mainly an effect of COVID-induced inflammation in patients, see [37]. Our results also demonstrated that age had a significant and positive association with the course of COVID so the older the patient, the more severe the disease. Again, these results align with previous research findings [2, 38, 39]. It was suggested that older adults may be more vulnerable to the effects of COVID-19 due to their weaker immune systems [40]. It is important to emphasize, however, that the results of our path analyses suggest that the association between age and COVID-19 severity is primarily mediated by long-term health issues. Consequently, healthy seniors may have a lower risk of severe COVID-19 than younger individuals with chronic health problems.

### Influence of time elapsed since infection on health and performance

As time has passed since the infection, there was a slight improvement in the health status of individuals (Tau = −0.032, *P* = .025). However, other monitored parameters remained largely unchanged, with the exception of a marginal shortening in reaction times in the respective performance test (Tau = −0.027, *P* = .057). A certain tendency toward improvement was also observed in the reaction times achieved in the Stroop test (Tau = −0.018, *P* = .192). Our findings, as it regards the persistence of some of the symptoms years beyond the initial COVID-19 infection, are consistent with those from university students [2], which demonstrated that the effects of COVID on health and performance could persist for several years, even though the participants were young (mean age 21.8) and none required hospitalization during their infection. That study reported that specific effects on mental health, reaction times, and particularly fatigue, may even begin to deteriorate again in the third year after infection, following an initial period of improvement. Our current study, concluded earlier in 2022, did not include individuals who had been infected for more than 2 years; hence, we could not observe a potential reversal in the trend of reaction time improvements in the third year post-infection. However, it is crucial to acknowledge that both studies were cross-sectional rather than longitudinal, and the experimental designs did not permit a clear distinction between the effects of time elapsed since infection and the potential effects of the viral variant that had caused the infection.

### Implications of COVID-19 vaccination status

Our research further identified a significant positive association between vaccination and both intelligence and precision. It is more likely that vaccination represents an outcome, rather than a driving factor, in these observed relationships. PA showed that intelligence had a strong positive effect on education and education in turn had a positive effect on the probability of being vaccinated. These two associations might have resulted in the observed relation between vaccination and intelligence. It must be reminded, however, that memory had also a strong effect on education despite no association between memory and vaccination was observed. It is, of course, possible that intelligence, but not memory, had also a direct effect on the probability of being vaccinated (standard PA cannot show the direction of arrows). Age was positively associated with vaccination, both directly (Tau = 0.06) and indirectly, through the mediation effect of long-term sickness. This finding suggests that older individuals were more likely to get vaccinated, possibly due to their higher perceived vulnerability to COVID, including the higher risk of mortality and serious COVID-related complications. Our study also found that older subjects had a lower probability of acquiring infection but a higher risk of a more severe course of COVID.

### Further observations from the study

In addition, our results demonstrated that a significantly higher percentage of men (93.95%) compared to women (90.71%) were vaccinated against COVID. This may reflect the influence of the information about COVID-19 risk factors in the news on the public as men are found and reported to be more vulnerable to COVID-19 [41], this may have encouraged them to get vaccinated more readily. However, in the population under study, men reported a less severe course of COVID than women.

We also discovered that vaccinated individuals had a significantly lower likelihood of being diagnosed with COVID-19 (49.05%) compared to nonvaccinated ones (67.24%), which potentially points to the role of vaccine-acquired immunity. However, it is highly likely that individuals who protect themselves against COVID-19 through vaccination also employ other protective measures such as mask-wearing and social distancing [42]. Therefore, it is unclear what the proximal cause of the lower infection risk in vaccinated participants is.

### Strengths and limitations

The study’s main strength was its large number of participants. In the majority of studies published so far, the number of participants has been considerably lower, or the cognitive performance of participants was assessed based on their subjective ratings rather than objective performance tests [43]. Additionally, our study leveraged a natural cohort of Internet users, ensuring a diverse representation of subjects across varying degrees of COVID-19 severity, in contrast to many prior studies that primarily focused on severe cases. An additional advantage of the current study was that participants were not informed in advance that one of the aims was to investigate the effects of COVID-19 and vaccination on health and performance. During recruitment and on the informed consent webpage, participants were told that the study would examine which biological and psychological factors influenced their performance test scores and moral attitudes. Questions about COVID-19 were placed toward the end of the 40–60 min questionnaire, after all performance tests, to avoid any conscious or unconscious distortion of results resulting from participants’ subjective opinions about the positive and negative effects of COVID-19 or vaccination on health and cognition (this procedure was approved in advance by the IRB). Another strength of the study was that participants were not given any rewards for their participation, which reduced the likelihood of “professional questionnaire fillers” or bots taking part.

The main limitation of the study is that the participants were self-recruited, and therefore, they do not represent a typical Czech population. Curious, altruistic people with interests in science were clearly overrepresented in the sample. Therefore, caution must be exercised when attempting to generalize the findings. Additionally, the physical health of the participants was self-reported and not examined by a medical professional. Although the study asked specific questions about the participants’ health and calculated health indices based on their responses, subjective factors such as illness anxiety and overall psychological attitudes might have influenced the resulting indices.

The strength of the observed effects, which is the fraction of total variability in focal variables attributed to COVID or vaccination, may seem relatively low. For instance, the partial Kendall Tau of −0.042 for the effect of severe COVID on the participants’ performance in the memory test corresponds to Cohen’s f of 0.06, which is typically categorized as a small effect (but not negligible). Small effect sizes are typical for this type of study, as test performance can be influenced by several factors, including the motivation of the participants and the precision, reliability, and reproducibility of the tests.

## Conclusions

Our study reveals that the after-effects of COVID-19 persist for several months, even in individuals with mild symptoms cases. This underscores the importance of factoring in protection against the disease’s long-term negative impact on cognitive performance when evaluating the costs and benefits of preventive measures, such as vaccination. We found that, besides age, sex, and long-term health conditions, education plays a vital role in reducing the risk of SARS-CoV-2 infection and severe COVID-19. The positive effect of education on reducing the risk of SARS-CoV-2 infection and severe COVID-19 is likely universal, and may also extend to reducing the risk of other diseases, enhancing the likelihood of vaccination and adherence to other preventive measures, thereby indirectly benefiting overall public health. Therefore, prioritizing and accelerating educational initiatives is imperative, forming an integral part of global public health strategies, crucial for both developing and developed nations alike.

## Conflicts of interest statement

The authors declare no competing interests.

## Funding 

This research was supported by Czech Science Foundation, grant number 22-20785S. Our sponsor had no involvement in the study design, the collection, analysis, and interpretation of data, the writing of the report, or in the decision to submit the article for publication.

## Authors’ contributions

Jaroslav Flegr (Conceptualization [equal], Formal analysis [equal], Funding acquisition [equal], Investigation [equal], Methodology [equal], Project administration [equal], Supervision [equal], Writing—original draft [equal], Writing—review & editing [equal]), and Ashkan Latifi (Formal analysis [equal], Investigation [equal], Validation [equal], Writing—original draft [equal], Writing—review & editing [equal])

## Data Availability

All data are available in the public repository figshare https://doi.org/10.6084/m9.figshare.22586446.v1.
